# Interdisciplinary Approaches to the Phenomenology of Auditory Verbal Hallucinations

**DOI:** 10.1093/schbul/sbu003

**Published:** 2014-06-07

**Authors:** Angela Woods, Nev Jones, Marco Bernini, Felicity Callard, Ben Alderson-Day, Johanna C. Badcock, Vaughan Bell, Chris C. H. Cook, Thomas Csordas, Clara Humpston, Joel Krueger, Frank Larøi, Simon McCarthy-Jones, Peter Moseley, Hilary Powell, Andrea Raballo, David Smailes, Charles Fernyhough

**Affiliations:** ^1^Centre for Medical Humanities, School of Medicine, Pharmacy and Health, Durham University, Durham, UK;; ^2^Lived Experience Research Network, Chicago, IL;; ^3^Department of English Studies, Durham University, Durham, UK;; ^4^Centre for Medical Humanities, Department of Geography, Durham University, Durham, UK;; ^5^Department of Psychology, Durham University, Durham, UK;; ^6^School of Psychology, University of Western Australia, Crawley, Australia;; ^7^Institute of Psychiatry, Psychosis Studies, King’s College London, London, UK;; ^8^Department of Theology and Religion, Durham University, Durham, UK;; ^9^Department of Anthropology, University of California San Diego, San Diego, CA;; ^10^Department of Sociology, Philosophy and Anthropology, University of Exeter, Exeter, UK;; ^11^Department of Psychology: Cognition and Behaviour, University of Liège, Liège, Belgium;; ^12^ARC Centre of Excellence in Cognitions and Its Disorders, Department of Cognitive Science, Macquarie University, Sydney, Australia;; ^13^Centre for Medical Humanities, Department of English Studies, Durham University, Durham, UK;; ^14^Department of Mental Health and Pathological Addiction, AUSL Reggio Emilia, Reggio Emilia, Italy

**Keywords:** auditory verbal hallucinations, phenomenology, interdisciplinarity, research collaboration, psychosis

## Abstract

Despite the recent proliferation of scientific, clinical, and narrative accounts of auditory verbal hallucinations (AVHs), the phenomenology of voice hearing remains opaque and undertheorized. In this article, we outline an interdisciplinary approach to understanding hallucinatory experiences which seeks to demonstrate the value of the humanities and social sciences to advancing knowledge in clinical research and practice. We argue that an interdisciplinary approach to the phenomenology of AVH utilizes rigorous and context-appropriate methodologies to analyze a wider range of first-person accounts of AVH at 3 contextual levels: (1) cultural, social, and historical; (2) experiential; and (3) biographical. We go on to show that there are significant potential benefits for voice hearers, clinicians, and researchers. These include (1) informing the development and refinement of subtypes of hallucinations within and across diagnostic categories; (2) “front-loading” research in cognitive neuroscience; and (3) suggesting new possibilities for therapeutic intervention. In conclusion, we argue that an interdisciplinary approach to the phenomenology of AVH can nourish the ethical core of scientific enquiry by challenging its interpretive paradigms, and offer voice hearers richer, potentially more empowering ways to make sense of their experiences.

## Introduction

The term “voice-hearing,” or auditory verbal hallucination (AVH), typically refers to hearing a voice or other sound in the absence of an external stimulus. The apparent simplicity of this mainstream definition belies the diversity of the experiences it names. Writing at the turn of the last century in what would become one of psychiatry’s most important textbooks, Emil Kraepelin^1^ described in detail the kinds of auditory hallucinations reported by patients suffering from what he called “dementia praecox.” Eschewing the relatively dry, flat language of the clinic, the patients spoke of:

“resonant voices,” “organ voices,” “voices of conscience,” “voices which do not speak with words,” “false voices,” “abortive voices,” an “inner feeling in the soul,” an “inward voice in the thoughts,” something “between hearing and foreboding,” “the brain talk[ing],” “voices in the whole body,” “murmurings and natural spirit-voices,” “underground voices from the air,” “telephone gossip,” “good voices,” and “whispering voices from the whole of mankind.”

How are we to make sense of these descriptions, which are echoed in the narratives of voice hearers today?^2^ Can conventional perception-centered definitions of AVHs, and even the mainstream metaphor of “hearing voices,” do justice to their complexity? How should we understand hallucinatory experiences within the arc of an individual’s life, and across different clinical and nonclinical populations? With the etiology and underlying mechanisms of AVHs a matter of ongoing research and debate, experience itself remains an important area of investigation.^3,4^

Given that AVHs are privy and particular to the individual, no research can avoid making decisions about the kinds of data and analytical tools that are to be considered sufficiently robust when it comes to investigating these most complex of scientific objects. In cognitive science, psychology, and psychiatry, empirical studies of AVH phenomenology typically utilize standardized scales in order to assess particular aspects of experience within a given population.^3,5–8^ However, while the validity, reproducibility, reliability, and foci of particular scales and measures are central to their scientific and clinical evaluation,^9^ hallucinations research can arguably benefit from more sustained engagement with the philosophical, epistemological, and theoretical issues underpinning such investigations.

One of the greatest contributions that might be made by the humanities and social sciences to the study of AVHs is in offering methods through which to conceptualize, delimit, identify, elicit, and analyze the so-called “subjective” data that form such a central component of this research. Whether or not it is addressed explicitly, all research inevitably has to negotiate what voice-hearing experiences denote, how they can be extracted from or identified in the fabric of an individual’s life, how they change over time, and if and how an AVH or voice is differentiated from other “normal” and “anomalous” forms of inner experience. Other important questions include: to what extent does representing and reporting AVHs—particularly in light of different cultural frameworks and available terms and constructs—impact upon the nature of that experience? And how might the relationship between the person experiencing the AVH and the person researching it be best conceptualized (not least in situations in which that person is the one and the same)? There are rich bodies of literature located in and across phenomenological psychiatry, anthropology, sociology, theology and religious studies, literary studies, history, medical humanities, and “mad studies”/service user led research that have much to offer vis-à-vis these questions. This literature can open up a variety of frameworks, methods, and analytical tools through which psychiatric researchers might reconsider what it is that they study when they study AVHs, and how they might go about acquiring and analyzing that data.

Recognizing that psychiatry has a long history of engagement with other disciplinary perspectives, the first sections of this article outline the purpose and value of an interdisciplinary approach to the phenomenology of AVH. Following Aboelela et al,^10^ we understand interdisciplinary research to involve collaboration between researchers from different disciplines “that links or integrates theoretical frameworks from those disciplines, uses study design and methodology that is not limited to any one field, and requires the use of perspectives and skills of the involved disciplines throughout multiple phases of the research process.” Durham University’s “Hearing the Voice” project is an example of an interdisciplinary approach to the study of AVH. With respect to phenomenology, such an approach would utilize rigorous and context-appropriate methodologies to analyze a wide range of first-person accounts of AVH at 3 contextual levels: (1) cultural, social, and historical; (2) experiential (in relationship to changes in the structure of experience); and (3) biographical (in relationship to the arc of an individual’s life). In the final sections we suggest 3 potential benefits for voice hearers, clinicians, and researchers: (1) informing the development and refinement of subtypes of hallucinations within and across diagnostic categories; (2) “front-loading” research in cognitive neuroscience; and (3) suggesting new possibilities for therapeutic intervention. Our focus is not on the wider contribution of the humanities and social sciences to the study of mental (ill) health and clinical practice; more narrowly, we seek to show how work in the humanities and social sciences can help us better understand the experience of hearing voices.

## Three Strengths of an Interdisciplinary Approach

### AVHs in Cultural, Social, and Historical Context

Has hearing voices always been part of human experience? The fact that “hallucination” itself is a recent^11^ and contested^12^ term raises the question of whether experiences so named have been the same across time, place, and culture. Anthropological studies of psychosis and schizophrenia have challenged the view that culture is of minor relevance to understanding AVH,^13–16^ showing that “local theory of mind—the features of perception, intention, and inference that the community treats as important—and local practices of mental cultivation will affect both the kinds of unusual sensory experiences that individuals report and the frequency of those experiences.”^17^ If the complexity of the relationship between culture and hallucinatory experience is to be adequately theorized and empirically investigated, researchers must make use of tools and disciplinary approaches which do not simply reduce “culture” to a one-dimensional variable (for which country of residence frequently functions as a proxy). Instead, as cultural psychiatrists and others^18,19^ have argued, ethnographic and qualitative approaches have a vital role to play in investigating the ways in which communities interpret, legitimize, support, and even produce different voice-hearing experiences.

Recognizing that mainstream biological psychiatry constitutes one such (albeit powerful) community can bring medical understandings of hallucination into dialog with other explanatory frameworks. Religious and spiritual accounts of voice hearing—which take seriously the meaning of voices and their origin in a realm beyond empirical study—call into question distinctions between “pathological” and other kinds of AVH. For example, recent anthropological studies of Pentecostal and charismatic Christian groups have found that members of these communities not infrequently report hearing the voice of God out loud and in external space, in ways they find spiritually encouraging and which are not associated with evidence of mental illness.^20,21^ Data collected through narrative and semistructured interviews, quantitative hallucination and absorption scales, ethnographic observation, and the study of theological and religious traditions, are here used to build an in-depth picture of voice-hearing experiences regarded as acceptable and even desirable within these Christian communities. As well as their intrinsic value (in contributing to our collective knowledge about the complexities of human experience), studies such as these also have instrumental value in clinical and research contexts (eg, in informing future taxonomies of AVH, as discussed below).

Of course, complex, multidimensional accounts of voice hearing are not confined to the present, so an interdisciplinary approach to the phenomenology of AVH must recognize its own historical specificity by taking into account the dis/continuities between contemporary accounts of voice hearing and those reported in earlier historical periods. Hearing the voice of God in the Judeo-Christian tradition again provides a useful case study. For the theologian studying scripture, as for the historian working with medieval Miracula, investigating the voices and visions of the past requires an understanding of the languages, practices, and beliefs which are reflected in and constituted by particular social, cultural, and political environments, as well as embedded in particular linguistic conventions. The voices heard by religious figures such as Ezekiel and Joan of Arc have been attributed to mental disorder by commentators, often in ways which reflect ignorance of the historical and textual worlds from which they speak to us.^22^ Given that scholarly engagement with the past is mediated primarily through textual forms, the act and status of writing becomes inextricable from the question of experience. Medieval mystic Margery Kempe’s experience of hearing voices was recorded by an amanuensis, which means that her “first-person account” might say as much about the process of transcription as about “experience” itself. Reading historical texts involves not only translation but interpretation: asking questions about why a text was written, how it was produced, the context of dissemination, its “social logic,” and even the reasons for its survival.^23^ The expert analysis of these texts provides insight into a wider realm of hallucinatory experiences as well as the sophisticated schemas for differentiating voice hearing that existed prior to its medicalization. From 16th century Spanish Carmelites such as St John of the Cross^24^ to contemporary charismatic Chicagoans,^21^ theologians, anthropologists, and historians have shown that the need to exercise discrimination in relation to voices—both literal and metaphorical—continues to be important to Christians, as to others, in the present day. Multiplying the frameworks through which voice-hearing experiences can be identified and interpreted, interdisciplinary approaches can offer new perspectives on the shared and nonshared features of these heterogeneous experiences, and so avoid circular arguments about what are to be counted as “pathological” and “non-pathological” voices.

### AVHs and the Structure of Experience

Phenomenology (literally, “the science of appearances”) is a philosophical movement which seeks to reflect upon the basic structures of experience—ie, experience from the first-person perspective of the subject—and to understand how these basic structures give our experience of the world and ourselves its formal coherence.^25^ For phenomenologists, this first-person emphasis entails a consideration of topics such as intentionality, self-awareness, temporality, embodiment, spatiality, agency, and intersubjectivity. Phenomenology has enjoyed a fruitful interaction with psychiatry and psychopathology for over a century, with phenomenological psychiatrists and psychologists (among them Jaspers, Minkowski, Blankenburg, Fuchs, Sass, Parnas, and Stanghellini) as well as nonclinical philosophers (including Gallagher, Zahavi, and Ratcliffe) making pioneering contributions to the analysis and theorization of psychosis.^26–32^

Following Husserl, there are generally thought to be 3 phases at the heart of phenomenological methodology: an initial bracketing of taken-for-granted assumptions or judgments about the cause, normality, or reality of what is experienced (including diagnostic or etiological considerations), which enables the investigator to focus on the character of the experience itself; discerning the prototypical features of the experience, its essence, or eidos, and generating descriptions that account for these features; finally, assessing the adequacy and fit of these descriptions and, when necessary, subjecting them to further elaboration and refinement. In a clinical context, a central part of this process involves focusing on the character and meaning of the person’s experience from their perspective, and continually submitting resultant descriptions to intersubjective scrutiny. Phenomenologists thus seek to analyze the specific structure of subtle qualitative changes in perceptual, proprioceptive, and intersubjective experience that might underlie psychopathological phenomena.^32^

Despite empirical findings corroborating a continuity in the severity of AVHs among clinical and nonclinical subjects^33^ studies have not yet addressed whether there is a corresponding continuum of phenomenology.^34,35^ Philosophical phenomenology (PP) is a methodology well suited to the investigation of subtle but potentially highly significant differences between the voice-hearing experiences of clinical and nonclinical individuals,^7^ and between different diagnostic groups including severe anxiety, military posttraumatic stress disorder, depression, and schizophrenia spectrum disorders.^36^ The auditory quality of voices is a case in point. Although descriptive psychopathologists have long recognized the nonauditory thought-like quality of many otherwise “psychotic” voices,^37^ contemporary measures and clinical interviews generally assume that self-reported voices are both literally auditory and identifiable in terms of auditory characteristics. Bracketing this assumption and analyzing the variety of ways in which voices are “heard” (as reported so clearly by Kraepelin’s patients quoted above) could illuminate differences which in turn have the potential to revise existing empirical measures. PP is also especially well placed to address hallucinatory complexities which are less frequently analyzed, such as the boundaries between externalized or externally located “thoughts” and literally “auditory” hallucinations, and the relationship between auditory and/or verbal aspects of hallucinations and their tactile, visual, affective, nonverbal, and/or somatic aspects.^38^ Should voice hearing be approached as a unitary, static, crystallized phenomenon, or as dynamic and subject to change over time?^39^ Do AVHs sometimes or always occur against the backdrop of a more encompassing transformation in one’s sense of reality? Through the rigorous analysis of individuals’ self-reports PP can identify fine-tuned structural differences and variances in how AVHs are experienced in relation to a person’s being in the world.^40–42^

### AVHs in Biographical Context

The narrative and biographical context(s) in which AVHs develop and are sustained have served as a key point of departure for many clinical and psychotherapeutic approaches to voices and psychosis, including psychoanalytic/psychodynamic therapies, existential and narrative therapies, trauma-informed care, and techniques developed within the international Hearing Voices Movement.^43–48^ Across these approaches, life events and internal struggles are viewed not only as significant causal contributors to the onset of AVHs, but also as major influences on their content, phenomenological form and structure, and degree of associated distress or disability.^44,49,50^ At the same time, and often in close proximity to these therapeutic approaches to voices, social scientists and humanities scholars have developed diverse models through which to understand and analyze the overarching concepts of “narrative” and “biography” and to consider what exactly constitutes the “context” of a life. While longitudinal psychiatric research typically makes some attempt to contextualize changes in symptomatology or functional disability,^51,52^ narrative biographical interviewing techniques might instead attempt to explore the subjective impact of factors identified as important by the participant in light of their own personal history.^53^ Narratological theories, finally, can highlight the importance of everyday storytelling in the processing and communication of life events and experiences, and the limitations imposed when opportunities to tell certain kinds of stories are constricted by cultural norms or sociopolitical factors.^54^ In the context of psychosis, a breakdown in the ability to tell integrated stories about the self has been implicated in the genesis of psychopathology^55^ and cultivated storytelling therefore underpins innovative work in narrative therapy.^56,57^

We emphasize that such methods extend beyond more familiar qualitative mental health research techniques such as thematic analysis^58^ or brief Interpretative Phenomenological Analysis.^59^ More critical and in-depth analytic methodologies seek to move past cross-sectional themes and categories and devise new ways to understand how conceptual frameworks, available cultural scripts, and biographical and embodied experiences might help structure and constrain both the subjective experience and communicated phenomenological form of AVHs.^60^ Much available qualitative mental health research remains committed to—and arguably constrained by—the categories and empirical methods currently dominant in mainstream schizophrenia research; we want to emphasize here, instead, the benefits of analytic frameworks that attempt to understand how language, narrative and embodied experience can both structure experience over time and provide potential tools for healing.

Complementing approaches from phenomenological psychiatry, sophisticated narrative and biographical approaches focus on the ways in which individuals negotiate their experiences of perceptual, affective, cognitive, and/or interpersonal change within specific social and cultural contexts. In addition, they help draw attention to the divergent and heterogeneous longitudinal trajectories of individuals with AVHs, including full recovery, periods of remission, deterioration, or more complex and varied changes in the nature, content, and valence of symptoms over time. Notably, much creative research and writing on narrative has been conducted by service users/those with lived experience,^61^ who have offered different accounts of what recovery might mean^62,63^ and whose work thereby demands that we attend more carefully to some of the standard categories (eg, “chronicity”) that are commonly used, often as shorthands, in psychiatric research on AVHs. Working collaboratively to refine these categories is an important task for future researchers. Narrative and biographical approaches may also help to link work in socioenvironmental epidemiology, clinical phenomenology and outcomes, for instance by suggesting or ruling out potential confounds or third variables, and connecting neurodevelopmental and epigenetic changes with subjective experience.

## Benefits to Clinical Research and Practice

### Informing Development and Refinement of Subtypes

One potential benefit of the approach advocated here is the development of a more accurate and nuanced phenomenological portrait of AVHs and the foregrounding of the radical phenomenological heterogeneity of the experience. Delineating new facets of AVHs challenges contemporary neurocognitive models of AVHs to account for the phenomena as they are, and not merely idealized, simplified, or partial versions of the experience.^64^

As we have argued, historical texts can constitute a valuable alternative “lens” through which to identify overlooked phenomenological aspects of voice hearing.^65^ For example, in his detailed taxonomy of hallucinations,^24^ St John of the Cross stressed the density of meaning of some voice-hearing experiences (instances in which voices may be experienced as communicating much more than simply what the words say).^65^ This resonates with and could inform the development of contemporary models of inner speech^66^ (especially those proposing a specific condensed variety which loses most of the acoustic and structural qualities of external speech and approaches the state of “thinking in pure meanings” described by Vygotsky^67^). Contemporary concretization of the term “hearing voices” has also led to the neglect of experience of “soundless voices” reported by St John of the Cross and across the centuries, including in the work of pioneering psychiatrists Bleuler,^68^ Kraepelin,^1^ and Jaspers.^26^ Such overlooked experiences are likely to offer important clues into the types of mentation that may form the raw material of AVHs, and remind us that the data of experience must be prioritized over existing theoretical accounts.^69^

Finally, the rich and detailed phenomenological descriptions we have argued for here should also aid the extension of the contemporary approach of using cluster analytic techniques on the phenomenological properties of AVHs to identify subtypes.^8,70^ This is particularly important, as identification of AVH subtypes offers the potential for tailored clinical interventions for specific subtypes of voice-hearing experiences.^35,36^

### “Front-loading” Cognitive-Neuroscientific Research

Given the National Institute of Mental Health’s shift to neuropsychiatric domains in place of categorical diagnoses or diagnostically specific symptoms, the exigency of addressing translational continuities and discontinuities between phenomenology and underlying biological change seems clear.^71^ Most experimental and correlational research continues to depend on self-report measures and tentative assumptions concerning the reliability of self-report. Neuroimaging studies, for instance, depend on self-report measures in order to categorize subjects experiencing or not experiencing AVHs, as well as on accurate “real-time” self-report within the scanner. More finely tuned phenomenological distinctions, including spatial location, loudness, and subjective reality, have proven important in understanding differential functional and structural alterations.^72–74^ Conversely, unquestioned assumptions about AVH phenomenology can potentially lead to the false aggregation or summation of potentially neuropathologically distinct phenomena and risk compromising the validity of the constructs used to study AVHs.

Research on the relationship between “auditory false perceptions” and AVH is a case in point. One commonly used cognitive paradigm used to study AVHs is the “signal detection” task, in which participants are asked to indicate with a button press whether they heard a voice in a burst of white noise. Findings suggest that people who experience AVHs are more likely to report that they heard a voice when no voice was present. It has therefore been suggested that these “auditory false perceptions” are, to some extent, analogous to “true” voice-hearing experiences^75^ (a finding supported by neuroimaging evidence suggesting similar brain activations during false alarm responses and AVHs).^76^ However, the extent of this analogy remains unclear: what do people “experience” when they make a false alarm response? Although data suggest that voice hearers show a decisional bias toward responding “yes,” it is also unclear what drives this bias—eg, are false alarm responses accompanied by a feeling of non–self-generation and alien-ness, as some AVHs are?^77^ Would different kinds of AVHs, such as those described as “hypervigilance” hallucinations,^78^ be more analogous to false alarm responses?

Researchers must decide how best to make the link between the “phenomenon” of voice hearing and the specific aspects of brain, body, or behavior under investigation, and how the relevant aspects of experience should be identified and used within experimental contexts. Advocating an interdisciplinary approach, philosopher Shaun Gallagher has urged a “front-loaded phenomenology,” whose guiding idea is to incorporate insights from phenomenological philosophy into experimental design.^79^ Philosophically informed phenomenological characterizations of AVH are notable for their richness and complexity; while selection from and even simplification of these accounts will be required in order to enable sufficient control over the many variables being studied, phenomenologically inspired studies of self-disturbances^80^ suggest this might be a fruitful direction for cognitive neuroscientific research into AVH.

### Enhancing Therapeutic Practice

Just as an interdisciplinary approach to the phenomenology of AVH can open up new ways of approaching taxonomies of voice hearing and the design of empirical studies, so too can the humanities, arts, and social sciences—in foregrounding the complexity, multidimensionality, and affective qualities of hallucinatory experiences—offer practical resources for individuals and clinicians seeking to understand and alleviate the distress which can accompany them. In this section we show how the narrative arts (cinema and literature), the tools of narrative and linguistic analysis, and approaches which take seriously the spiritual significance of voices, can offer valuable resources in the therapeutic encounter.

Phenomenological psychiatrist Karl Jaspers^26^ claimed that the process of developing a psychotic symptom could be subject to objective or causal explanation (erklären) but not an ability to empathically grasp the subjective experience and know what it is like (verstehen). While art has explored madness over millennia, the fact that the formation of psychotic symptoms is an extended process suggests that narrative arts are some of the best placed to help communicate the subjective experiences which Jaspers famously claimed were “ununderstandable.” As a medium including both sensory and narrative aspects, cinema can comprehensively characterize complex psychological disturbances and help make them empathically available to the spectator.^81^ Masterpieces of cinema d’auteur (as directed by Bergman, Buñuel, and Cronenberg, eg) offer a “vision from within,” capturing aspects of psychosis in all its existential and interpersonal impact. Enigmatic and sometimes impenetrable subjective experiences can be depicted on screen in several ways: either by observation—where the viewer sees the behavior of someone experiencing AVHs; by insightful representation—where the viewer sees the world as the voice hearer does, in full knowledge that the experiences are not shared by others; by first-person representation—where the viewer is not aware, at least at the time, that the experiences are not shared; or by analogy—where psychosis-like experiences are accurately represented but not identified as such in the story.^81^

Literature provides another invaluable repertoire for studying the distinguishing features of AVHs and problematizing the alleged commonality of the experience. The techniques of narrative fiction enable the reader to experience a “transparency” in accessing multiple fictional minds.^82^ For example, through the fiction of Virginia Woolf,^83^ Samuel Beckett,^84^ and Hilary Mantel^85^ we can gain access to shifting perspectives on the content of hallucinatory experience; its evolution across time; and its embodied, interpersonal, and social dimensions. Immersion in a fictional world can also trigger in the reader a complex empathetic experience, allowing the reader to enact a simulative experience of the event.^86^ The value of fiction here is underscored by the relative paucity of longitudinal studies of AVH and the difficulties faced by empirical researchers in accessing and measuring subtle changes within individuals’ experiences.

Voices have the distinction of being symptoms which speak: ie, symptoms which often appear as language. Literary theory and in particular narratology can provide powerful, if underrecognized, tools with which to investigate self-reports of AVHs. First-person accounts are often shaped as narratives in which the voice is described as a speaking character (ie, personalized with name, intentions, temper). Complementing psychological studies of persons’ relationship with their voices,^87^ a narratological analysis of such talkative acts can highlight structural commonalities or specific features such as the voice’s narrative distance and point of view (speaking in first person, second person, third person, or a plural “we”); spatial and temporal indexing (here, there, this, that time, and so on); and the consonant or dissonant narration (in terms of content, timeline, ideology, information) the voice produces in relationship to the voice hearer. Furthermore, if the voice hearer as interpreter of her voices can also be considered as a reader, contemporary cognitive narratology can shed light on how—in this interpretive activity – she is able to construct a “continuing-consciousness frame”^88^ for this voice or fill the gaps in the voice’s narration. Analyses of linguistic style and word use^89^ likewise can provide simple but powerful tools to assist voice hearers to track for themselves how they are thinking and communicating in their daily lives (eg, via emails and diaries) both with voices and with others. While these are important goals in their own right, the potential for literary and linguistic methods to fine-tune taxonomies of voice hearing and so feed into translational research also warrants further exploration.

Within the experience of those who are diagnosed as suffering from mental disorder, spiritual and religious themes arise frequently. Among a group of people diagnosed as suffering from schizophrenia, 60% reported that they found religion helpful in coping with their experience of hearing voices.^90^ Psychiatrists are less likely to be religious than their patients,^91^ and so the potential for pathological interpretation of religious experience—thus denying its importance as a coping resource—is significant. In fact, first-hand accounts of voice hearing, from those of medieval mystics^92^ to contemporary users of mental health services^93,94^ suggest that people can and do distinguish between “spiritual” voices and voices which they themselves consider to be a manifestation of illness. Similarly, some professionals have suggested systematized criteria by which distinctions between spiritual and pathological voices can be made^95^ whereas others have suggested that a range of unusual experiences—including divine or mystical voices and paranormal phenomena—might all in fact be a part of a realm of the “borderline” or “transliminal.”^96^

Interdisciplinary approaches to phenomenology as outlined here can have practical benefits in helping to develop better patient-therapist relationships. It has been argued that, without a good patient-therapist relationship, the efficacy of cognitive behavioral therapy (CBT) is reduced, and that therapist empathy is important in fostering this relationship.^97^ This claim is supported by the reports of patients with a diagnosis of schizophrenia^98^ and by data showing that patient ratings of therapist empathy are a predictor of therapeutic alliance in CBT for psychosis.^99^ If the core feature of empathy is “the ability to understand the patient’s situation, perspective and feelings”^100^ it seems likely that interdisciplinary approaches to phenomenology would be helpful in communicating these features of a patient’s world to clinicians who might otherwise struggle to empathize with hallucinatory experiences.^101^

## Conclusion

This article has argued for the value of an interdisciplinary approach to the phenomenology of AVH to complement, challenge, enrich, and extend mainstream hallucination research. AVHs do not exist except in context: the context of human consciousness, of an individual’s life, and as made meaningful within the explanatory frameworks available in particular places and historical periods. While it can be extremely fruitful to take AVHs “aside” and examine them in isolation and from different disciplinary perspectives, we also must not forget that AVHs are part of the gestalt of the person. Empirical psychological and psychiatric accounts of AVH phenomenology can be enriched, we have argued, by an interdisciplinary approach which utilizes the robust methodologies of the humanities and social sciences to fully realize this contextual complexity, inextricable as it is from experience itself. This has significant potential benefits for the refinement of AVH subtypes, informing empirical work, and enhancing therapeutic practice.

Interdisciplinary approaches from the humanities and the social sciences demand that we all—no matter what our own expertise or topic of research—need to examine which sources, frameworks, and models we explicitly or implicitly foreground in the production, analysis, and dissemination of data relating to the experience of voice hearing. As well as offering new ways of working with and thinking through what the “subjective” might mean in research on AVH, these literatures raise difficult questions concerning which disciplines, which theoretical perspectives, and which kinds of expertise have most authority in determining how concepts are defined, how the category of “voice-hearing” is delineated from other experiences, and which questions are most pressing for researchers to answer. The challenges of interdisciplinary research are practical as well as philosophical. Work that endeavors to cross epistemic, disciplinary, discursive, and professional-cultural divides can be difficult and time consuming to produce, and harder still to disseminate. Yet multiperspectival approaches can nourish the ethical core of scientific enquiry by challenging prevailing interpretive paradigms and showing that these are, as with every paradigm, culturally and historically grounded.^102^ They also offer rich possibilities for voice hearers in making sense of their experiences outside the relatively narrow frameworks of conventional psychiatric frameworks. As indicated in the supplementary table, the real challenge now is to work toward ways in which an interdisciplinary approach that foregrounds and values multiple forms of expertise—professional and experiential—can be fully integrated into mainstream psychiatric and hallucination research.

## Supplementary Material

Supplementary material is available at http://schizophreniabulletin. oxfordjournals.org.

## Funding

Wellcome Trust Strategic Award (WT098455MA) to A.W., C.C., S.M.-J., and C.F.

## Supplementary Material

Supplementary Data

## References

[1] Kraepelin E Clinical Psychiatry. Vol 7 New York: Scholars’ Facsimiles & Reprints; 1981

[2] Romme M Escher S Dillon J Corstens D Morris M Living with Voices: Fifty Stories of Recovery. Ross-on-Wye: PCCS Books; 2009

[3] Larøi F Sommer IE Blom JD The characteristic features of auditory verbal hallucinations in clinical and nonclinical groups: state-of-the-art overview and future directions. Schizophr Bull. 2012;38:724–733 22499783 10.1093/schbul/sbs061 PMC3406519

[4] Waters F Aleman A Fernyhough C Allen P Report on the inaugural meeting of the International Consortium on Hallucination Research: a clinical and research update and 16 consensus-set goals for future research. Schizophr Bull. 2012;38:258–262 22223735 10.1093/schbul/sbr181 PMC3283162

[5] Bell V Raballo A Larøi F Assessment of hallucinations. In: Larøi F Aleman A, eds. Hallucinations: A Practical Guide to Treatment and Management. Oxford: Oxford University Press; 2010

[6] Larøi F How do auditory verbal hallucinations in patients differ from those in non-patients? Front Hum Neurosci. 2012;6:25 22375112 10.3389/fnhum.2012.00025 PMC3282922

[7] McCarthy-Jones S Krueger J Larøi F Broome M Fernyhough C Stop, look, listen: the need for philosophical phenomenological perspectives on auditory verbal hallucinations. Front Hum Neurosci. 2013;7:127 23576974 10.3389/fnhum.2013.00127 PMC3620561

[8] McCarthy-Jones S Trauer T Mackinnon A Sims E Thomas N Copolov DL A new phenomenological survey of auditory hallucinations: evidence for subtypes and implications for theory and practice. Schizophr Bull. 2014;40:231–235 23267192 10.1093/schbul/sbs156 PMC3885292

[9] ICHR. New Assessment Methods for Hallucinations Working Group. Durham, UK: 2nd International Consortium on Hallucination Research; September 2013

[10] Aboelela SW Larson E Bakken S Defining interdisciplinary research: conclusions from a critical review of the literature. Health Serv Res. 2007;42:329–346 17355595 10.1111/j.1475-6773.2006.00621.x PMC1955232

[11] Berrios G Marková IS The construction of hallucination: history and epistemology. In: Blom JD Sommer IEC, eds. Hallucinations: Research and Practice. London: Springer; 2011:55–71

[12] Romme M Escher S Making Sense of Voices: A Guide for Mental Health Professionals Working with Voice-Hearers. London: Mind Publications; 2000

[13] Jenkins JH Barrett RJ Schizophrenia, Culture, and Subjectivity: The Edge of Experience. Cambridge: Cambridge University Press; 2004

[14] Littlewood R Dein S Did Christianity lead to schizophrenia? Psychosis, psychology and self reference. Transcult Psychiatry. 2013;50:397–420 23749775 10.1177/1363461513489681 PMC4107833

[15] Fabrega H Jr On the significance of an anthropological approach to schizophrenia. Psychiatry. 1989;52:45–65 2648433 10.1080/00332747.1989.11024429

[16] Luhrmann T Padmavati R Hearing voices in San Mateo, Accra and Chenai. Culture, mind, and brain: emerging concepts, methods, applications; October 20, 2012; UCLA.

[17] Luhrmann T Hallucinations and sensory overrides. Annu Rev Anthropol. 2011;40:71–85

[18] Larøi F Luhrmann TM Bell V Culture and hallucinations: overview and future directions. Schizophr Bull. 2014;40(suppl 4):S213–S220 24936082 10.1093/schbul/sbu012 PMC4141319

[19] Kirmayer LJ Ban L Cultural psychiatry: research strategies and future directions. Adv Psychosom Med. 2013;33:97–114 23816867 10.1159/000348742

[20] Dein S Littlewood R The voice of God. Anthropol Med. 2007;14:213–228 10.1080/13648470701381515 27268394

[21] Luhrmann T When God Talks Back: Understanding the American Evangelical Relationship With God. New York: Knopf; 2012

[22] Cook CCH Psychiatry in scripture: sacred texts and psychopathology. The Psychiatrist. 2012;36:225–229

[23] Spiegel GM History, historicism, and the social logic of the text in the middle ages. Speculum. 1990;65:59–86

[24] Kavanaugh K Rodriguez O The Collected Works of St John of the Cross. Washington, DC: Institute of Carmelite Studies; 1991

[25] Moran D Introduction to Phenomenology. London: Routledge; 2000

[26] Jaspers K General Psychopathology. 7th ed Manchester: Manchester University Press; 1963

[27] Sass LA Self and world in schizophrenia: three classic approaches. Philos Psychiat Psychol. 2001;8:251–270

[28] Broome MR Harland R Owen GS Stringaris A The Maudsley Reader in Phenomenological Psychiatry. Cambridge: Cambridge University Press; 2013

[29] Fuchs T Corporealized and disembodied minds: a phenomenological view of the body in melancholia and schizophrenia. Philos Psychiat Psychol. 2005;12:95–107

[30] Stanghellini G Fuchs T One Century of Karl Jaspers’ General Psychopathology. Oxford: Oxford University Press; 2013 International Perspectives in Philosophy & Psychiatry.

[31] Fulford K Davies M Gipps R The Oxford Handbook of Philosophy and Psychiatry. Oxford: Oxford University Press; 2013

[32] Parnas J Sass LA Zahavi D Rediscovering psychopathology: the epistemology and phenomenology of the psychiatric object. Schizophr Bull. 2013;39:270–277 23267191 10.1093/schbul/sbs153 PMC3576163

[33] Hill K Linden D Hallucinatory experiences in non-clinical populations. In: Jardri R Cachia A Thomas P Pins D, eds. The Neuroscience of Hallucinations. New York: Springer; 2013:21–41

[34] McCarthy-Jones S Fernyhough C The varieties of inner speech: links between quality of inner speech and psychopathological variables in a sample of young adults. Conscious Cogn. 2011;20:1586–1593 21880511 10.1016/j.concog.2011.08.005

[35] Johns LC Kompus K Connell M Auditory verbal hallucinations in persons with and without a need for care. Schizophr Bull. 2014;40 (suppl 4):S255–S264 24936085 10.1093/schbul/sbu005 PMC4141313

[36] McCarthy-Jones S Thomas N Strauss C Better than mermaids and stray dogs? Subtyping auditory verbal hallucinations and its implications for research and practice. Schizophr Bull. 2014;40(suppl 4):S275–S284 24936087 10.1093/schbul/sbu018 PMC4141311

[37] Tuttle GT Hallucinations and illusions. *Am J Psychiat* 1902;58:443–467

[38] Waters F Collerton D ffytche D Visual hallucinations in the psychosis spectrum and comparative information from neurodegenerative disorders and eye disease. Schizophr Bull. 2014;40(suppl 4):S233–S245 24936084 10.1093/schbul/sbu036 PMC4141306

[39] Raballo A Larøi F Murmurs of thought: phenomenology of hallucinatory consciousness in impending psychosis. Psychosis: Psychological, Social and Integrative Approaches. 2011;3:163–166

[40] Romdenh-Romluc K Merleau-Ponty’s account of hallucination. Eur J Philos. 2009;17:76–90

[41] Thomas P Bracken P Leudar I Hearing voices: a phenomenological-hermeneutic approach. Cogn Neuropsychiat. 2004;9:13–23 10.1080/13546800344000138 16571572

[42] Stanghellini G Cutting J Auditory verbal hallucinations: breaking the silence of inner dialogue. Psychopathology. 2003;36:120–128 12845282 10.1159/000071256

[43] Read J Ross CA Psychological trauma and psychosis: another reason why people diagnosed schizophrenic must be offered psychological therapies. J Am Acad Psychoanal Dyn Psychiatry. 2003;31:247–268 12722898 10.1521/jaap.31.1.247.21938

[44] Longden E Corstens D Escher AD Romme M Voice hearing in a biographical context: a model for formulating the relationship between voices and life history. Psychosis: Psychological, Social and Integrative Approaches. 2012;4:224–234

[45] Fenton WS Evolving perspectives on individual psychotherapy for schizophrenia. Schizophr Bull. 2000;26:47–72 10755669 10.1093/oxfordjournals.schbul.a033445

[46] Roberts G Narrative and severe mental illness: what place do stories have in an evidence-based world? Adv Psychiatric Treatment. 2000;6:432–441

[47] Corstens D Longden E McCarthy-Jones S Waddingham R Thomas N. Emerging perspectives from the Hearing Voices Movement: implications for research and practice. Schizophr Bull. 2014;40(suppl 4):S285–S294 24936088 10.1093/schbul/sbu007 PMC4141309

[48] Thomas N Hayward M Peters E Psychological therapies for auditory hallucinations (voices): current status and key directions for future research. Schizophr Bull. 2014;40(suppl 4):S202–S212 24936081 10.1093/schbul/sbu037 PMC4141318

[49] Lysaker PH Lysaker JT Psychosis and the disintegration of dialogical self-structure: Problems posed by schizophrenia for the maintenance of dialogue. Br J Med Psychol. 2001;74 Part 1:23–33 11314900

[50] Pérez-Álvarez M García-Montes JM Vallina-Fernández O Perona-Garcelán S Cuevas-Yust C New life for schizophrenia psychotherapy in the light of phenomenology. Clin Psychol Psychother. 2011;18:187–201 20677182 10.1002/cpp.716

[51] Ayesa-Arriola R Rodríguez-Sánchez JM Pérez-Iglesias R The relevance of cognitive, clinical and premorbid variables in predicting functional outcome for individuals with first-episode psychosis: a 3 year longitudinal study. Psychiatry Res. 2013;209:302–308 23403293 10.1016/j.psychres.2013.01.024

[52] Harrow M Jobe TH How frequent is chronic multiyear delusional activity and recovery in schizophrenia: a 20-year multi-follow-up. Schizophr Bull. 2010;36:192–204 18617485 10.1093/schbul/sbn074 PMC2800138

[53] Wengraf T Qualitative Research Interviewing: Biographic Narrative and Semi-Structured Methods. London: Sage; 2001

[54] Frank A Letting Stories Breathe:A Socio-Narratology. Chicago: University of Chicago Press; 2010

[55] Lysaker PH Lysaker JT Narrative Structure in Psychosis: Schizophrenia and Disruptions in the Dialogical Self. Theory Psychol. 2002;12:207–220

[56] Lysaker PH Lancaster RS Lysaker JT Narrative transformation as an outcome in the psychotherapy of schizophrenia. Psychol Psychother. 2003;76:285–299 14577894 10.1348/147608303322362505

[57] Madigan S Narrative Therapy. Washington, DC: American Psychological Association; 2011

[58] Braun V Clarke V Using thematic analysis in psychology. Qualitat Res Psychol. 2006;3:77–101

[59] Smith JA Flowers P Larkin M Interpretative Phenomenological Analysis: Theory, Method and Research. London: Sage; 2009

[60] Blackman L Immaterial Bodies: Affect, Embodiment, Mediation. London: Sage; 2012

[61] Neila ST Pricea J Pitta L Working together: Service Users and researchers in psychosis research. Psychosis:Psychological, Social and Integrative Approaches. 2013;5:306–316

[62] Pitt L Kilbride M Nothard S Welford M Morrison AP Researching recovery from psychosis: a user-led project. The Psychiatrist. 2007;31:55–60

[63] Woods A Romme M McCarthy-Jones S Dillon J Escher S Editorial: voices in a positive light. Psychosis:Psychological, Social and Integrative Approaches. 2013;5:213–215

[64] McCarthy-Jones S Hearing Voices: The Histories, Causes and Meaning of Auditory Verbal Hallucinations. Cambridge: Cambridge University Press; 2012

[65] Jones SR Re-expanding the phenomenology of hallucinations: lessons from sixteenth-century Spain. Mental Health, Religion and Culture. 2010;13:187–208

[66] Fernyhough C Alien voices and inner dialogue: towards a developmental account of auditory verbal hallucinations. New Ideas in Psychology. 2004;22:49–68

[67] Vygotsky LS Thinking and Speech. New York: Plenum Press; 1987

[68] Bleuler E Dementia Praecox or The Group of Schizophrenias. New York: International Universities Press; 1950

[69] Leuder I Thomas P Voices of Reason, Voices of Insanity: Studies of Verbal Hallucinations. London: Routledge; 2000

[70] Garwood L Dodgson G Bruce V McCarthy-Jones S. A preliminary investigation into the existence of a hypervigilance subtype of auditory hallucination in people with psychosis. Behav Cogn Psychother. 2013 Aug 20:1–11. [Epub ahead of print]. 10.1017/S1352465813000714 23962410

[71] Ford JM Morris SE Hoffman RE Studying hallucinations within the NIMH RDoC framework. Schizophr Bull. 2014;40 (suppl 4):S295–S304 24847862 10.1093/schbul/sbu011 PMC4141312

[72] Plaze M Paillère-Martinot ML Penttilä J “Where do auditory hallucinations come from?”–a brain morphometry study of schizophrenia patients with inner or outer space hallucinations. Schizophr Bull. 2011;37:212–221 19666833 10.1093/schbul/sbp081 PMC3004180

[73] Raij TT Valkonen-Korhonen M Holi M Therman S Lehtonen J Hari R Reality of auditory verbal hallucinations. Brain. 2009;132:2994–3001 19620178 10.1093/brain/awp186 PMC2768657

[74] Vercammen A Knegtering H Bruggeman R Aleman A Subjective loudness and reality of auditory verbal hallucinations and activation of the inner speech processing network. Schizophr Bull. 2011;37:1009–1016 20164122 10.1093/schbul/sbq007 PMC3160225

[75] Barkus E Smallman R Royle N Barkus C Lewis S Rushe T Auditory false perceptions are mediated by psychosis risk factors. Cogn Neuropsychiatr. 2011;16:289–302 10.1080/13546805.2010.530472 21113827

[76] Barkus E Stirling J Hopkins R McKie S Lewis S Cognitive and neural processes in non-clinical auditory hallucinations. Br J Psychiatry Suppl. 2007;51:s76–s81 18055942 10.1192/bjp.191.51.s76

[77] Nayani TH David AS The auditory hallucination: a phenomenological survey. Psychol Med. 1996;26:177–189 8643757 10.1017/s003329170003381x

[78] Dodgson G Gordon S Avoiding false negatives: are some auditory hallucinations an evolved design flaw? Behav Cogn Psychother. 2009;37:325–334 19371459 10.1017/S1352465809005244

[79] Gallagher S Zahavi D The Phenomenological Mind: An Introduction To Philosophy Of Mind And Cognitive Science. 2nd ed London: Routledge; 2012

[80] Hur JW Kwon JS Lee TY Park S The crisis of minimal self-awareness in schizophrenia: A meta-analytic review. Schizophr Res. 2014;152:58–64 24055201 10.1016/j.schres.2013.08.042

[81] Raballo A Larøi F Bell V Humanizing the clinical gaze: movies and the empathic understanding of psychosis. Fam Med. 2009;41:387–388 19492180

[82] Cohn D Transparent Minds: Narrative Modes for Presenting Consciousness in Fiction. Princeton: Princeton University Press; 1983

[83] Woolf V Mrs Dalloway. London: Penguin; 1992

[84] Beckett S Three Novels: Molloy, Malone Dies, the Unnamable. New York: Grove Press; 2009

[85] Mantel H Beyond Black. London: Fourth Estate; 2005

[86] Goldman AL Simulating Minds: The Philosophy, Psychology, and Neuroscience of Mindreading. Oxford: Oxford University Press; 2006

[87] Benjamin LS Is chronicity a function of the relationship between the person and the auditory hallucination? Schizophr Bull. 1989;15:291–310 2749190 10.1093/schbul/15.2.291

[88] Palmer A Fictional Minds. Lincoln: University of Nebraska Press; 2004

[89] Pennebaker JW The Secret Life of Pronouns. What Our Words Say About Us. New York: Bloomsbury Press; 2011

[90] Mohr S Gillieron C Borras L Brandt PY Huguelet P The assessment of spirituality and religiousness in schizophrenia. J Nerv Ment Dis. 2007;195:247–253 17468685 10.1097/01.nmd.0000258230.94304.6b

[91] Cook CCH The faith of the psychiatrist. Mental Health, Religion & Culture. 2011;14:9–17

[92] Stanley L The Book of Margery Kempe. Kalamazoo, MI: Medieval Institute Publications; 1996

[93] Michalak EE Yatham LN Kolesar S Lam RW Bipolar disorder and quality of life: a patient-centered perspective. Qual Life Res. 2006;15:25–37 16411028 10.1007/s11136-005-0376-7

[94] McCarthy-Jones S Waegeli A Watkins J Spirituality and hearing voices: considering the relation. Psychosis. 2013;5:247–258 24273597 10.1080/17522439.2013.831945 PMC3827668

[95] Menezes A Jr Moreira-Almeida A Religion, spirituality, and psychosis. Curr Psychiatry Rep. 2010;12:174–179 20425277 10.1007/s11920-010-0117-7

[96] Clarke I Psychosis and spirituality: the discontinuity model. In: Clarke I, ed. Psychosis and Spirituality: Consolidating the New Paradigm. 2nd ed Oxford: Wiley-Blackwell; 2010:101–114

[97] Beck AT Rush AJ Shaw BF Emery G Cognitive Therapy of Depression. New York: Guilford; 1979

[98] Fox V Empathy: the wonder quality of mental health treatment. Psychiatric Rehab J. 2000;23:292–293

[99] Evans-Jones C Peters E Barker C The therapeutic relationship in CBT for psychosis: client, therapist and therapy factors. Behav Cogn Psychother. 2009;37:527–540 19728901 10.1017/S1352465809990269

[100] Coulehan JL Platt FW Egener B “Let me see if i have this right.”: words that help build empathy. Ann Intern Med. 2001;135:221–227 11487497 10.7326/0003-4819-135-3-200108070-00022

[101] Chadwick PDJ Birchwood MJ Trower P Cognitive Therapy for Delusions, Voices and Paranoia. Chichester: Wiley; 1996

[102] Schrader S Illuminating the heterogeneity of voices in a multiple perspectives research paradigm. Psychosis:Psychological, Social and Integrative Approaches. 2013;5:216–225

