# Supplementary material for: Interdisciplinary Approaches to the Phenomenology of Auditory Verbal Hallucinations
Source: Schizophr Bull. 2014 Jun 7;40(Suppl 4):S246–54. doi: 10.1093/schbul/sbu003 (PMC4141308; doi:10.1093/schbul/sbu003)
Supplement: Supplementary Data [file supp_40_Suppl-4_S246__index.html]

Interdisciplinary Approaches to the Phenomenology of Auditory Verbal Hallucinations — Interdisciplinary Approaches to the Phenomenology of Auditory Verbal Hallucinations — Interdisciplinary Approaches to the Phenomenology of Auditory Verbal Hallucinations — Supplementary Data 

# Interdisciplinary Approaches to the Phenomenology of Auditory Verbal Hallucinations

## Supplementary Data

Data files

**Files in this Data Supplement:**

- Supplementary Data - Supplementary Data
