# Supplementary material for: Interdisciplinary Approaches to the Phenomenology of Auditory Verbal Hallucinations
Source: Schizophr Bull. 2014 Jun 7;40(Suppl 4):S246–54. doi: 10.1093/schbul/sbu003 (PMC4141308; doi:10.1093/schbul/sbu003)
Supplement: Supplementary Data [file supp_sbu003_Supplementary_Material.docx]

# Supplementary Material

| **Table 1: Practical Recommendations for Future Research**  1. The data produced by research are an outcome of the tools used to study AVHs. In order to develop a more accurate understanding of these phenomena, researchers should prioritise methodologies that can capture the heterogeneity of AVHs, their change over time and relationship to other aspects of experience.  2. Humanities scholars, social scientists, and researchers with lived experience of AVHs can make valuable contributions to study design and data analysis by utilizing robust methodologies to understand AVHs within experiential, biographical and cultural context.  3. Researchers studying AVHs should consider the full breadth of relevant disciplines when planning the make-up of research teams and seek meaningful collaborations with researchers and advocates with first-person experience of hallucinations.  4. Literary, cinematic and first-person representations of AVH can provide valuable ‘case studies’ for analysing the phenomenology of voice-hearing, as well as opportunities to enhance clinicians’ insight into these experiences. Narratological analysis of the ‘talkative acts’ of AVHs might yield important insights into sub-types with implications for translational research.  5. Translational mixed methods research should be considered with the explicit aim of integrating insights from rigorous qualitative and philosophical investigations of AVHs with on-going work in the neurosciences, developmental biopsychiatry, and epigenetics.  6. Clinical research projects on biopsychosocial intervention should likewise engage with work on the cultural and socio-political dimensions of recovery and individuals’ own exploration of the meaning of their experiences. |
| --- |
